# Severe Outbreak of *Saprolegnia* Spp. Infection in Spotted Snakehead (*Channa punctata*, Bloch 1793): Clinical Assessment, Histopathology, Haemato‐Biochemical Indices and Insights Into Therapeutic Effects

**DOI:** 10.1002/vms3.70473

**Published:** 2025-07-24

**Authors:** Md. Shaif Rahman, Md. Siddikur Rahman Sujon, Susmita Roy, Shamima Nasren, Tofael Ahmed Sumon, Sarker Md. Ibrahim Khalil, M. M. Mahbub Alam, Md. Abdullah Al Mamun

**Affiliations:** ^1^ Department of Fish Health Management Laboratory of Fish Diseases Diagnosis and Pharmacology Faculty of Fisheries Sylhet Agricultural University Sylhet Bangladesh; ^2^ Department of Fish Biology and Genetics Faculty of Fisheries Sylhet Agricultural University Sylhet Bangladesh

**Keywords:** *Channa punctata*, haemato‐biochemical indices, histopathology, saprolegniasis, Tanguar *haor*, therapeutic effects

## Abstract

Saprolegniasis, caused by *Saprolegnia* spp., is one of the most lethal oomycete infections affecting freshwater fish. This study investigated a severe fungal infection in *Channa punctata* (*n* = 150) fish obtained from a pen culture in the Tanguar *haor* region of the Sunamganj District. The infection was identified by its characteristic fluffy white appearance, with fungal hyphae detected in multiple organs. Diagnosis was confirmed as *Saprolegnia* spp. through clinical signs and wet mount examination. Infected fish exhibited notable symptoms, including drowsiness, head‐down floating, cloudy eyes, skin burns and deep lesions, with broken caudal fins in some cases. The infection prevalence was calculated at 85%. For further growth analysis, Czapek–Dox agar (CDA) medium was used to culture fungal hyphae. The average weight and length of the fish were 74.41 ± 1.32 g and 14.38 ± 0.84 cm, respectively. Subsequently, 120 infected fish were allocated into 4 treatment groups, each placed in glass aquaria (90 × 45 × 45 cm^3^) with 120 L of water and treated for a 10‐day experimental period. The groups included T1 (control), T2 (ivermectin, 2.5 ppm + NaCl, 2%), T3 (CuSO_4_ 1 ppm + Viodin 2.5 ppm) and T4 (aquarium heater set to 30°C + NaCl, 2%). Significant recovery was noted in T4, where the cotton‐like structures disappeared within 10 days. Haematological and biochemical indices also revealed significant differences between T1 and T4. Histopathological analysis identified several cellular abnormalities, such as granuloma formation, fungal hyphae presence, dermal degeneration, gill and liver haemorrhages, kidney necrosis and tubular degeneration and splenitis with intracellular oedema. Post‐treatment, histopathological anomalies were notably reduced in T4, with fewer fungal hyphae observed, whereas moderate alterations remained in T3 and T2 compared to the control group. Kaplan–Meier survival analysis indicated that *C. punctata* achieved the most favourable survival rate (*p* < 0.05) in T4 by combining elevated temperature with NaCl, resulting in a 70% survival rate, whereas the lowest survival rate of 23.33% was observed in T1. This study offers valuable insights for effective treatment and management of *Saprolegnia* spp. infections in *C. punctata*.

## Graphical abstract

Spotted snakehead (*Channa punctata*) naturally infected with *Saprolegnia* spp. were confirmed by clinical signs, wet mount examination and histopathology.

In the therapeutic trial using various chemotherapeutics, elevated temperature combined with 2% NaCl (T4) resulted in the highest survival rate (70%).

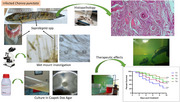

## 1 Introduction

Bangladesh is fortunate to have over 230 rivers, both majestic and charming, which contribute to its incredible water‐based wealth (Hasan et al. 2019). Due to abundant water resources, it has enormous opportunities for aquaculture. Almost every day in Bangladesh, the aquaculture sector is growing rapidly from the previous day. Among 260 freshwater species, including 55 families and 145 genera—overall, 58% of them are small indigenous species (SIS), which have a length not more than 25 cm or 9 in (Hossain 2010; Ali et al. 2016). SIS, a delicacy of Bangladeshi cuisine because of their protein content, play a vital role in the Bangladeshi diet, contributing to nutrition levels and preventing malnutrition of rural low‐income earners (Tacon and Metian 2013; Belton et al. 2014). These fish are rich in animal protein, fatty acids, vitamins and minerals, making them crucial for both economic and nutritional security not only for the poor but also for everyone in Bangladesh, a country which belongs to the global south (Mohanty et al. 2013; Faridullah et al. 2022). Taki fish (*Channa punctata*), also known as the spotted snakehead, are SIS, known as piscivorous, carnivorous, exhibiting cannibalistic behaviour and the ability to thrive in harsh environmental conditions. However, it's important to note that Taki fish, like other SIS, are also susceptible to diseases.

Disease is regarded to be the most significant constraint in SIS farming (van West 2006). Environmental stressors, such as poor water quality, malnutrition and overpopulation, can result in fish illnesses (Abhiman et al. 2021). Fish can be readily attacked by aquatic fungi like *Saprolegnia* spp., which is the most prevalent of the several fungi that are harmful to fish (Chauhan 2014). Fungal diseases, particularly saprolegniasis, are among the most significant disorders affecting the Egyptian fish culture and resulting in major financial losses (Eissa et al. 2013; Mostafa et al. 2020; Elameen et al. 2021). Fishes like *Catla catla*, *Cirrhinus mrigala*, *Labeo rohita* (Chauhan et al. 2014), *Heteropneustes fossilis*, *Mystus cavasius* (Mastan 2015), *Clarias batrachus* (Mastan and Ahmad 2018), *Cyprinus carpio* (Magray et al. 2021), *Channa marulius*, *Channa striata*, *Oreochromis niloticus*, *Oreochromis mossambicus* (Olufemi 1985), salmonids (Hussein et al. 2001), *Ictalurus punctatus*, *Anguilla anguilla* (Bruno 1999) and many more freshwater species are susceptible to this pathogen. When temperature is at its lowest during winter, autumn or in the early spring, normally *Saprolegnia* spp. can be found affecting a greater number of individuals (Hughes 1962; Elgendy et al. 2023). Cotton wool–like structure, broken dorsal and caudal fins, loss of appetite, abnormal swimming pattern, haemorrhages and skin ulceration are the main clinical signs of saprolegniasis in fish. Mass mortality can occur when a significant number of fish in a population are infected with pathogens, particularly at the final stage of their life cycle (Borisutpeth et al. 2009; Chauhan et al. 2014).

Investigation into the treatment of a variety of fungal infections and impacts on life in the water or the surroundings has received a lot of attention for many decades. Toxic chemicals that are used to treat saprolegniasis exhibit a teratogenic and carcinogenic impact in the tissues of fish (Schreier et al. 1996; van West 2006). Malachite green, formalin (Willoughby and Roberts 1992), 37% concentrated solution of formaldehyde (Schreier et al. 1996), hydrogen peroxide (Derksen et al. 1999; Howe et al. 1999), iodine‐free salt (NaCl), Bronopol (Pottinger and Day 1999) and a plethora of investigations have supported the administration and the application of boric acid, clotrimazole, potassium permanganate, copper nitrate, copper sulphate and medications like Pyceze (Pottinger and Day 1999) are used, even though many studies are being conducted to identify an alternative therapy for saprolegniasis due to the ban of malachite green application for treatment (Bly et al. 1997; Pottinger and Day 1999). In an extensive review, Elgendy et al. (2024) reported that alternative therapies—particularly those based on herbal extracts—have shown promising results in controlling saprolegniasis, offering safer and more eco‐friendly options for disease management in aquaculture.

Haemato‐biochemical indices are crucial in clinical diagnostics as they provide comprehensive insights into the health of a fish to diagnose, monitor and manage various diseases by assessing blood and biochemical markers (Mamun et al. 2022). Moreover, histopathology is crucial in fungal infection diagnosis, enabling the identification of fungal elements, assessment of tissue invasion and guiding targeted antifungal therapy based on specific pathogen characteristics (Sekhawat 2024). Therefore, the present research aimed to understand the infection caused by *Saprolegnia* spp. in snakehead fish, covering clinical signs, underlying causes, disease progression and potential management approaches through the application of chemicals that are environmentally safe and sustainable and are highly efficient in terms of economy, health and welfare of the fish.

## 2 Materials and Methods

### 2.1 Sampling Site and Layout of the Experiment

**FIGURE 1:**
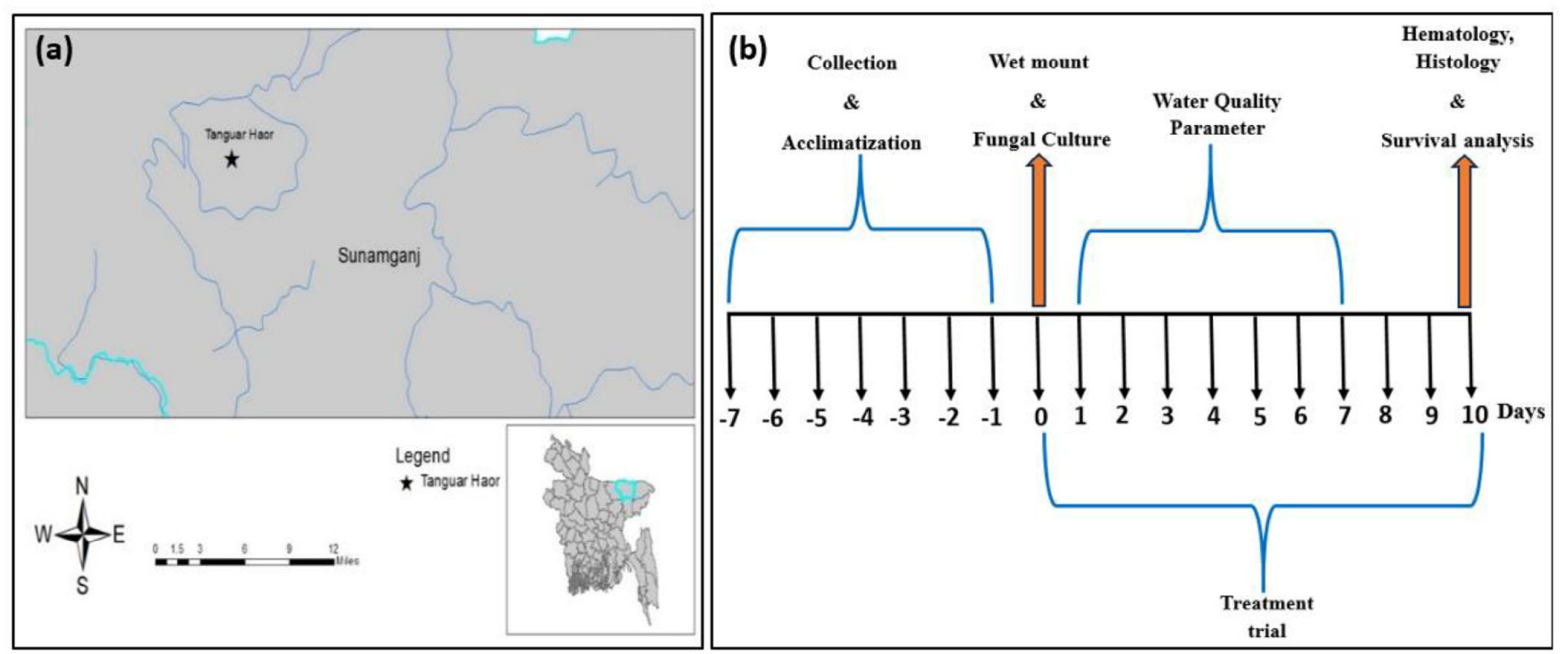
(a) Sampling site; (b) experimental layout illustrating the study timeline, with the first 7 days dedicated to the collection and acclimatization of *Channa punctata*, followed by a 10‐day treatment period. During the treatment phase, fungal culture, wet mount examination, histology, water quality parameters, haematology and survival analysis were conducted.

Fish were collected from Tanguar *haor* located in the Sunamganj District, Sylhet Division, Bangladesh (Figure 1). A total of 150 infected *C. punctata* specimens were collected directly from a pen culture system and transported to the Fish Disease Diagnosis and Pharmacology Laboratory at Sylhet Agricultural University, Bangladesh.

### 2.2 Experimental Design for Treatment Trial

A total of 120 *C. punctata* naturally infected with *Saprolegnia* were randomly selected from the initial stock and divided into four treatment groups (T1–T4). Each treatment group consisted of 3 replicate tanks, with 10 fish per tank (*n* = 30 per group). Randomization was performed using a lot‐drawing method. The sample size was based on logistical feasibility and previous studies with similar therapeutic trial designs. All groups, including the untreated control group, consisted of infected fish to assess treatment efficacy under uniform disease conditions. The treatments were applied for 10 days to evaluate the efficacy of different therapeutic agents in managing *Saprolegnia* spp. infections. Infected fish were transferred to aquaria (90 cm × 45 cm × 45 cm) and fed once daily with a commercial pelleted feed (36% crude protein, ACI Godrej Agro Pvt. Ltd.) at 3% of their body weight. The average body weight and length of the fish were 74.41 ± 1.32 g and 14.38 ± 0.84 cm, respectively. The therapeutic agents and materials used in this study included Acimec 1% Oral Solution Vet (Ivermectin BP 10 mg/mL; ACI Ltd., Bangladesh), copper(II) sulphate anhydrous, puriss. p.a. (Sigma‐Aldrich, Merck, Germany) and Viodin 10% Solution (Povidone‐Iodine USP 10 g/100 mL; Square Pharmaceuticals Ltd., Bangladesh). Aquarium salt (NaCl) was sourced locally from Bangladesh. Temperature regulation was achieved using a submersible aquarium heater (Shanghai, China). The therapeutic strategies used in the current experiment are outlined in Table 1. Water was partially renewed twice during the experimental period—on the third and seventh days. Approximately 30% of the total water volume was replaced each time, and the respective therapeutic agents were re‐administered according to the original dosages.

**TABLE 1:** Therapeutic agents and their dosages in different treatment trials.

SL no.	Treatment	Therapeutic agents
T1	Control	—
T2	Ivermectin + salt	2.5 ppm + 2%
T3	CuSO_4_ + Viodin^a^	1 + 2.5 ppm (3 mL/120 L)
T4	Temperature + salt	30°C + 2%

^a^ Viodin—Each 100 mL solution contains Povidone‐Iodine USP 10 g (3 mL of Viodin per 120 L of water = 2.5 ppm of Povidone‐Iodine).

### 2.3 Culture of *Saprolegnia* spp

Muscle tissues with ulcers were aseptically removed and cut into 1‐in^2^ blocks. Samples were taken from all groups (*n* = 3 per group) at 0 day post‐treatment trial. The blocks were cultured on Czapek–Dox agar (CDA) with ampicillin (100 units/mL) and oxolinic acid (100 units/mL) and incubated for 24–48 h at 25°C. Hyphal growth was observed using a light microscope (Carl Zeiss Primostar 3, Germany), and morphological characteristics were noted.

### 2.4 Wet Mount Preparation

**FIGURE 2:**
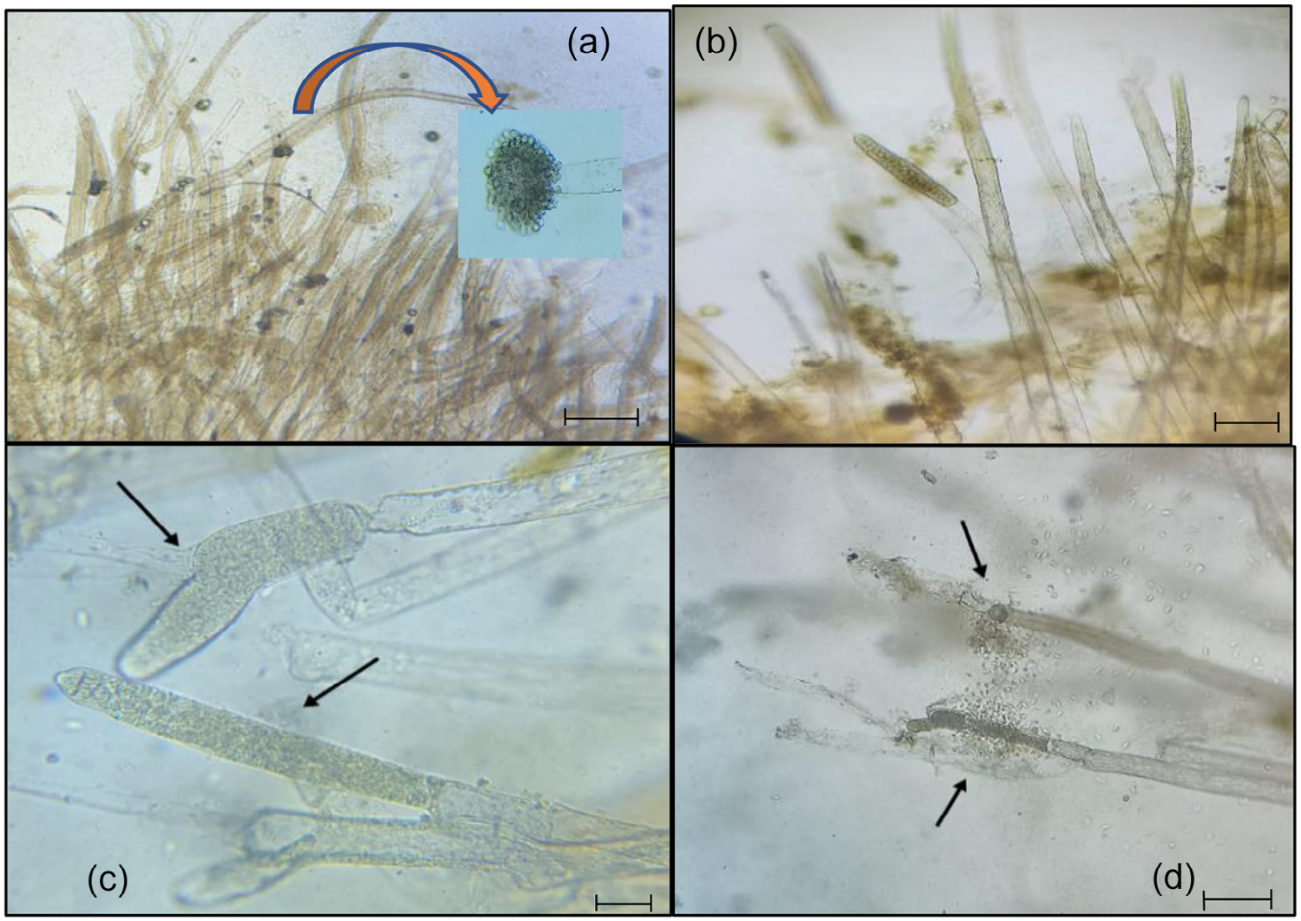
(a) Numerous fungal hyphae with developed oogonium (arrowhead, 10×; 200 µm). (b and c) Mature fungal hypha with zoospore (10×; 200 µm). (d) Regions with released zoospores (arrow) (10×; 200 µm).

Fluffy, cotton‐like samples were taken from the skin, head, gills and fins of fish in all groups (*n* = 3 per group) at 0 day post‐treatment trial. Samples were mounted on slides with water and covered for observation under 100× magnification, using immersion oil for clarity (Figure 2).

### 2.5 Water Quality Analysis

Water quality was monitored every alternate day using a Digital Multimeter (YSI Professional Plus multiparameter water quality meter, USA).

### 2.6 Haemato‐Biochemical Analysis

Three fish were sampled from each replication, resulting in a total of nine fish per treatment group except with the control group, where seven fish were alive and used for the haemato‐biochemical indices. They were anaesthetized with MS222 at a dose of 0.1 mL/L of water for haematological analysis. Blood was collected from the caudal peduncle using a 1 mm syringe pre‐rinsed with heparin (Rotexmedica, Trittau, Germany; 5000 IU/mL) and promptly transferred to tubes containing di‐potassium EDTA, following the method of Hurbee and Smith (2000). Haematological indices were measured using an automated haematology analyser (PE‐7010) in the Department of Physiology and Pharmacology, Faculty of Veterinary, Animal and Biomedical Sciences, Sylhet Agricultural University (SAU). Plasma samples were obtained by centrifuging the remaining blood at 5000 rpm for 15 min and stored at −20°C until further analysis. For biochemical parameter analysis, a clinical chemistry analyser (AGD‐2020) was used at the Laboratory of Fish Disease Diagnosis and Pharmacology, Faculty of Fisheries, SAU, with serum parameters assessed using kits from AGD Biomedicals Pvt. Ltd., Mumbai, India.

### 2.7 Histology Procedure

Skin, muscles, gills, liver, spleen and kidney samples were preserved in aqueous 10% neutral buffered formalin for 48–72 h for histological evaluation. After that, the tissue underwent standard processing to become paraffin blocks. Tissue fragments, cut to a thickness of 4–6 mm, were stained with haematoxylin and eosin (H&E) (Mamun et al. 2021). Histopathological changes were identified and assessed following the criteria established by Pacorig et al. (2022).

### 2.8 Statistical Analysis

The statistical analysis was done using Microsoft Excel and IBM SPSS Statistics version 26 for Windows. To assess the variability and central tendency of each variable, the standard deviation (SD) and average were estimated through a one‐way analysis of variance (ANOVA). To identify significant differences among the variables, Duncan's multiple range test was employed, considering a significance level of *p* < 0.05. For survival data, Kaplan–Meier analysis was applied, and group differences were assessed using the log‐rank (Mantel–Cox) test.

## 3 Results

### 3.1 Clinical Signs and Behavioural Observation

Aberrant movement and visible abnormalities throughout the body surface, eye and gills of 120 infected live fish were examined and documented. Infected fish showed behavioural abnormalities, including diminished appetite, operculum movement, impaired balance resulting in erratic movement, surface‐oriented swimming behaviour, scale detachment and haemorrhages among major organs and others.

### 3.2 Culture of *Saprolegnia* Spp

*Saprolegnia* spp. was seen as non‐septate, multinucleated mycelium with an uneven, white, transparent, velvety surface. These hyphae, with rounded tips, held the zoospores. The appearance of a cottony, white growth marked the zoospore colonization. By the second day, hyphae started growing from the culture medium and completely covered the plate by the fifth day.

### 3.3 Wet Mount Identification

The hyphae of the *Saprolegnia* spp. were found branching as well as rather thick. The major zoosporangia terminal on the main branch of the zoosporangium is cylindrical. Zoospores were typically spherical and motile.

### 3.4 Morphometric Features and Survival Rate

Table 2 provides an overview of the experimental groups, detailing the morphometric characteristics and survival rates of *C. punctata*.

**TABLE 2:** Overview of the experiment.

Group	No. of fish	Body features *Channa punctata*		10 days		Survival rate (%)
		Length (cm)	Weight (g)	Dead	Alive	
T1	30	14.00 ± 0.84^a^	75.70 ± 1.97^a^	23	7	23.33^a^
T2	30	13.82 ± 1.23^a^	78.35 ± 2.07^a^	16	14	46.67^b^
T3	30	15.03 ± 1.17^a^	71.22 ± 2.63^a^	14	16	53.33^b^
T4	30	14.67 ± 0.90^a^	72.39 ± 2.11^a^	9	21	70^c^

*Note*: Data are expressed as mean ± standard deviation (M ± SD); different superscript alphabets in each row are remarkably (*p* < 0.05) different.

### 3.5 Water Quality Parameter

The water quality parameters varied significantly (*p* < 0.05) across the treatment groups (T1–T4). T4 maintained the highest temperature (30°C) and lowest dissolved oxygen (5.67 ± 0.42 mg/L) compared to the other groups. DO levels in T1–T3 ranged from 7.23 to 7.36 mg/L. T3 had the highest pH (8.22 ± 0.11), whereas T1 had the lowest (7.48 ± 0.18) (*p* < 0.05). Ammonia levels were highest in T4 (5.05 ± 0.18) and lowest in T3 (3.14 ± 0.55) (*p* < 0.05). Nitrite levels showed no significant differences between groups (*p* > 0.05) (Table 3).

**TABLE 3:** Value of physicochemical parameters of the water in each treatment.

Parameter	T1	T2	T3	T4
Temp (°C)	25.43 ± 0.48^a^	25.33 ± 0.33^a^	25.49 ± 0.07^a^	30.00 ± 0.00^b^
DO (mg/L)	7.26 ± 0.09^b^	7.23 ± 0.20^b^	7.36 ± 0.26^b^	5.67 ± 0.42^a^
pH	7.48 ± 0.18^a^	8.13 ± 0.15^b^	8.22 ± 0.11^b^	8.17 ± 0.15^b^
NH_3_	4.20 ± 0.18^bc^	3.79 ± 0.25^ab^	3.14 ± 0.55^a^	5.05 ± 0.18^c^
Nitrite	0.35 ± 0.12^a^	0.35 ± 0.05^a^	0.33 ± 0.02^a^	0.25 ± 0.04^a^

*Note*: Values are expressed as means ± SD; significant differences among means in the same row, as determined by one‐way ANOVA followed by the Duncan's multiple range, are denoted by the absence of a common superscript letter (*p* < 0.05).

### 3.6 Haemato‐Biochemical Parameters

The haematological parameters of *C. punctata* infected with *Saprolegnia* spp. displayed distinct alterations across the treatment groups, as shown in Table 4. In the control group (T1), a significant increase (*p* < 0.05) was observed in white blood corpuscle (WBC), mean corpuscular volume (MCV), packed cell volume (PCV), mean corpuscular haemoglobin (MCH), neutrophils and monocytes compared to other treatments. Conversely, T1 showed significantly lower levels (*p* < 0.05) of haemoglobin (Hb), red blood corpuscle (RBC) and platelets than the treated groups. Serum analysis (Table 5) indicated that T1 had significantly higher values (*p* < 0.05) for serum glutamic pyruvic transaminase (SGPT), serum glutamic‐oxaloacetic transaminase (SGOT), AP and globulin. Meanwhile, T4 showed significantly elevated levels (*p* < 0.05) of albumin, total protein, high‐density lipoprotein (HDL), low‐density lipoprotein (LDL), cholesterol (CHO), triglycerides (TGs) and random blood sugar (RBS), aligning with its highest survival rate.

**TABLE 4:** Blood profile of *Channa punctata* across treatment groups.

Treatment				
Parameters	T1	T2	T3	T4
Hb (%) (g/dL)	10.47 ± 0.55^a^	11.4 ± 0.7^ab^	13.13 ± 1.30^b^	11.93 ± 1.68^ab^
RBC (m/UL)	2 ± 0.36^a^	2.35 ± 0.42^ab^	2.6 ± 0.41^ab^	2.93 ± 0.30^b^
WBC (×10^3^/mm^3^)	89.29 ± 7.15^c^	32.67 ± 4.07^a^	69.33 ± 2.55^b^	32.87 ± 1.99^a^
Neutrophils (%)	17.87 ± 0.94^b^	16.76 ± 0.38^b^	14.37 ± 0.21^a^	13.17 ± 1.04^a^
Lymphocytes (%)	68.8 ± 0.2^b^	72.27 ± 1.10^c^	73.57 ± 0.93^c^	65.93 ± 1.10^a^
Monocytes (%)	9.5 ± 0.43^b^	8.8 ± 0.3^b^	9.1 ± 0.4^b^	6.9 ± 1.01^a^
Eosinophils (%)	5.63 ± 0.64^a^	5.43 ± 0.40^a^	5.17 ± 0.32^a^	5 ± 0.2^a^
Basophils (%)	1 ± 0.57^ab^	1 ± 0.5^ab^	1.00 ± 0.5^a^	1.06 ± 0.11^b^
HCT/PCV (%)	41.6 ± 2.85^c^	31.93 ± 3.20^b^	25.90 ± 2.77 ^a^	36.86 ± 3.65^bc^
MCV	162.93 ± 29.17^b^	137.22 ± 11.09^ab^	130.83 ± 11.19^ab^	125.73 ± 2.15^a^
MCH (Pg)	44.91 ± 10.34^ab^	45.23 ± 5.75^ab^	68.08 ± 19.86^b^	41.37 ± 8.16^a^
MCHC	27.51 ± 2.79^a^	32.88 ± 1.57^a^	51.46 ± 10.66^b^	32.88 ± 7.73^a^
Platelet (10^3^/mm^3^)	25.68 ± 0.54^b^	23.27 ± 0.2^a^	22.64 ± 0.43^a^	29.01 ± 0.21^c^

*Note*: Values are expressed as means ± SEM, significant differences among means in the same row, as determined by one‐way ANOVA followed by the Duncan's multiple range, are denoted by the absence of a common superscript letter (*p* < 0.05).

Abbreviations: Hb, haemoglobin; MCH, mean corpuscular haemoglobin; MCHC, mean cell haemoglobin concentration; MCV, mean corpuscular volume; PCV, packed cell volume; RBC, red blood corpuscle; WBC, white blood corpuscle.

**TABLE 5:** Comparative serum biochemistry of *Channa punctata* under different treatments.

Parameter	T1	T2	T3	T4
SGPT (UL)	71.63 ± 0.58^d^	63.57 ± 1.15^c^	57.33 ± 2.08^b^	43.00 ± 1.73^a^
SGOT (UL)	837.00 ± 14.11^d^	430.00 ± 9.00^c^	194.13 ± 3.51^b^	95.33 ± 3.05^a^
AP	40.42 ± 1.52^c^	27.71 ± 2.08^a^	33.67 ± 2.52^b^	34.50 ± 3.21^bc^
Total protein (g/dL)	3.10 ± 0.10^a^	3.54 ± 0.20^b^	4.37 ± 0.10^c^	5.10 ± 0.79^d^
HDL (mg/dL)	55.66 ± 0.47^a^	61.27 ± 1.52^b^	68.00 ± 2.64^c^	75.00 ± 1.73^d^
LDL (mg/dL)	17.25 ± 1.43^a^	35.13 ± 1.58^b^	51.81 ± 1.25^c^	58.37 ± 1.53^d^
CHO (mg/dL)	85.71 ± 2.52^a^	91.34 ± 1.53^b^	123.17 ± 3.21^c^	146.19 ± 2.89^d^
TG (mg/dL)	79.67 ± 1.57^a^	88.21 ± 1.54^b^	116.00 ± 2.64 ^c^	140.53 ± 1.58^d^
RBS (mg/dL)	17.33 ± 2.08^b^	10.00 ± 1.06^a^	13.16 ± 1.53^ab^	138.17 ± 4.93^c^
Albumin (g/dL)	1.13 ± 0.15^a^	1.53 ± 0.11^a^	1.77 ± 0.11^b^	2.06 ± 0.06^c^
Globulin	0.94 ± 0.05^a^	1.30 ± 0.20^ab^	1.09 ± 0.21^a^	1.60 ± 0.36^b^
AG ratio	1.20 ± 0.22^a^	1.18 ± 0.47^b^	1.62 ± 0.57^b^	1.29 ± 0.79^c^

*Note*: Values are expressed as means ± SEM; significant differences among means in the same row, as determined by one‐way ANOVA followed by Duncan's multiple range, are denoted by the absence of a common superscript letter (*p* < 0.05).

Abbreviations: AG, albumin globulin; AP, alkaline phosphate; CHO, cholesterol; HDL, high‐density lipoprotein; LDL, low‐density lipoprotein; RBS, random blood sugar; SGOT, serum glutamic‐oxaloacetic transaminase; SGPT, serum glutamic pyruvic transaminase; TG, triglyceride.

### 3.7 Histological Alterations in Different Organs of *C. punctata*

#### 3.7.1 Histopathology of Skin

**FIGURE 3:**
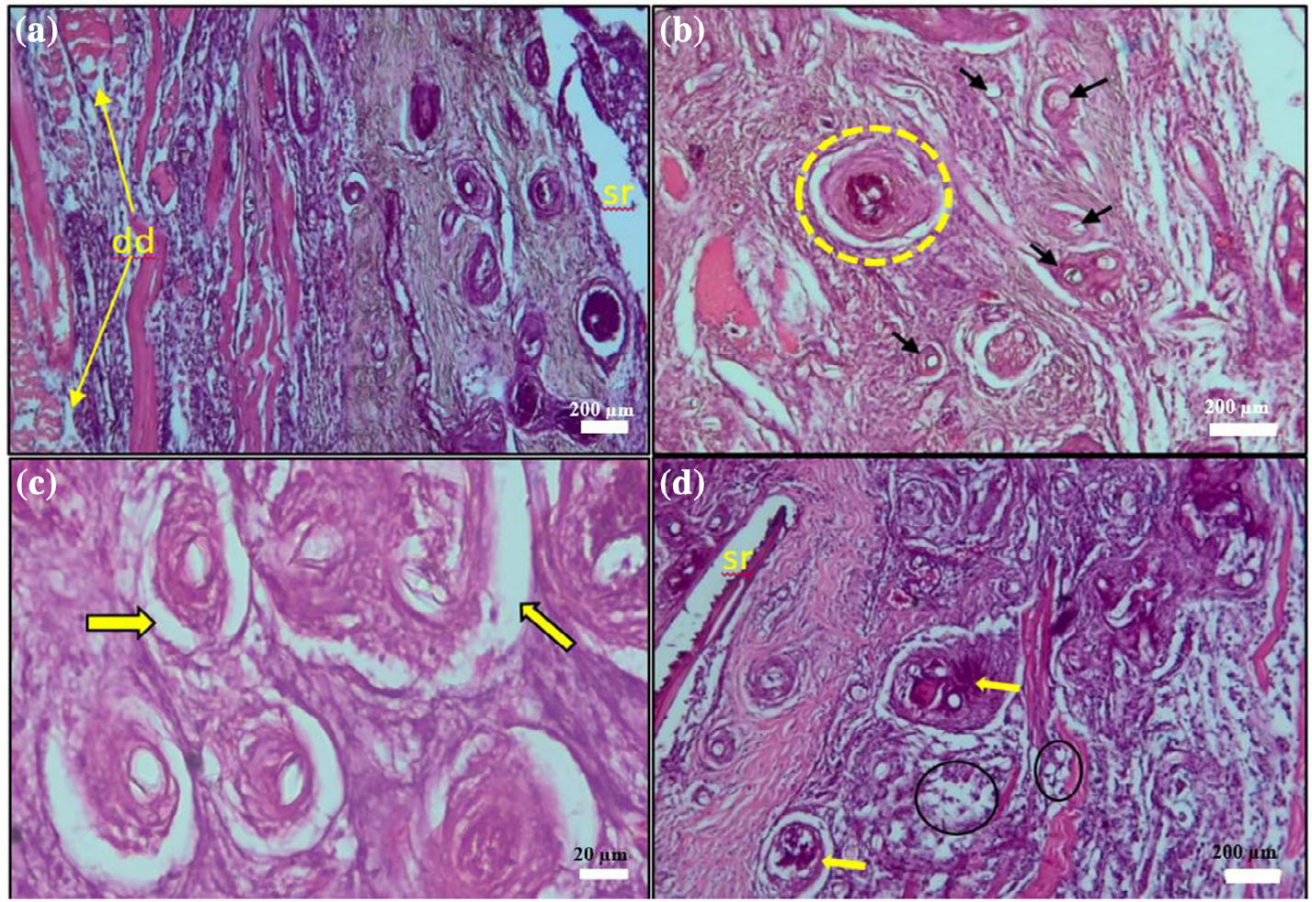
Histopathology of *Saprolegnia* spp. infected skin (a) dermal layer degeneration (dd) yellow arrow, skin rapture (sr); (b) mature granuloma with necrotic centre surrounded by fibroblast layers (yellow circle), vertical cross‐sectional fungal hyphae (black arrow); (c) a number of granulomata (arrow) surrounded by fibroblast layers in *Channa punctata*; (d) encapsulation fungi by foreign body type giant cells (circle) and a granuloma (arrow) surrounded by thick fibroblast layers (20–200 µm) (H&E).

Figure 3 highlights histological abnormalities in the skin, including thick fibroblast layers, fungal hyphae, dermal degeneration, necrosis and skin rupture. Notably, T4 showed a marked reduction in these abnormalities, eventually restoring normal skin structure (Table 6).

**TABLE 6:** Impact of treatment on the histopathological alteration of different organs in *Channa punctata*.

		Treatments			
Organ	Anomalies	T1	T2	T3	T4
Gill	Clubbing	+++	+++	+++	+
	Oedema	+++	++	++	+
	Hypertrophy	+++	+++	+	−
	Hyperplasia	+++	+++	++	−
	Lamellar fusion	++	+	++	−
Skin	Dermal layer degeneration	+++	++	+++	−
	Mature granuloma	++	−	+	−
	Fibroblast	+++	+	++	+
	Granuloma	+	−	−	++
	Fungal hyphae	+++	+++	++	−
Kidney	Glomerular shrinkage	+	++	+	−
	Vacuolation	+++	++	++	+
	TDDT	++	+	+	+
	TDPT	++	++	−	−
	Necrosis	+++	++	+	−
Liver	EIBS	+	++	++	+
	MMCs	++	+	−	−
	Haemorrhage	+++	+	++	+
	Blood vessel shrinkage	++	−	−	−
	Sinusoid dilation	+	++	+	+
Spleen	MMCs	+++	++	++	+
	Intracellular oedema	+++	+	−	−
	Raptured spleen cell	++	—	+	−
	Sinusoidal space	+++	+	++	−
	Necrotic eosinophil	++	++	−	−

*Note*: ‘−’, no pathological change; ‘+’, mild alteration; ‘++’, moderate alteration; ‘+++’, severe alteration.

Abbreviations: EIBS, erythrocyte infiltration into blood sinusoid; MMC, melanomacrophage centres; TDDT, tubular degeneration of distal tubule; TDPT, tubular degeneration of proximal tubule.

#### 3.7.2 Histopathology of Gill

**FIGURE 4:**
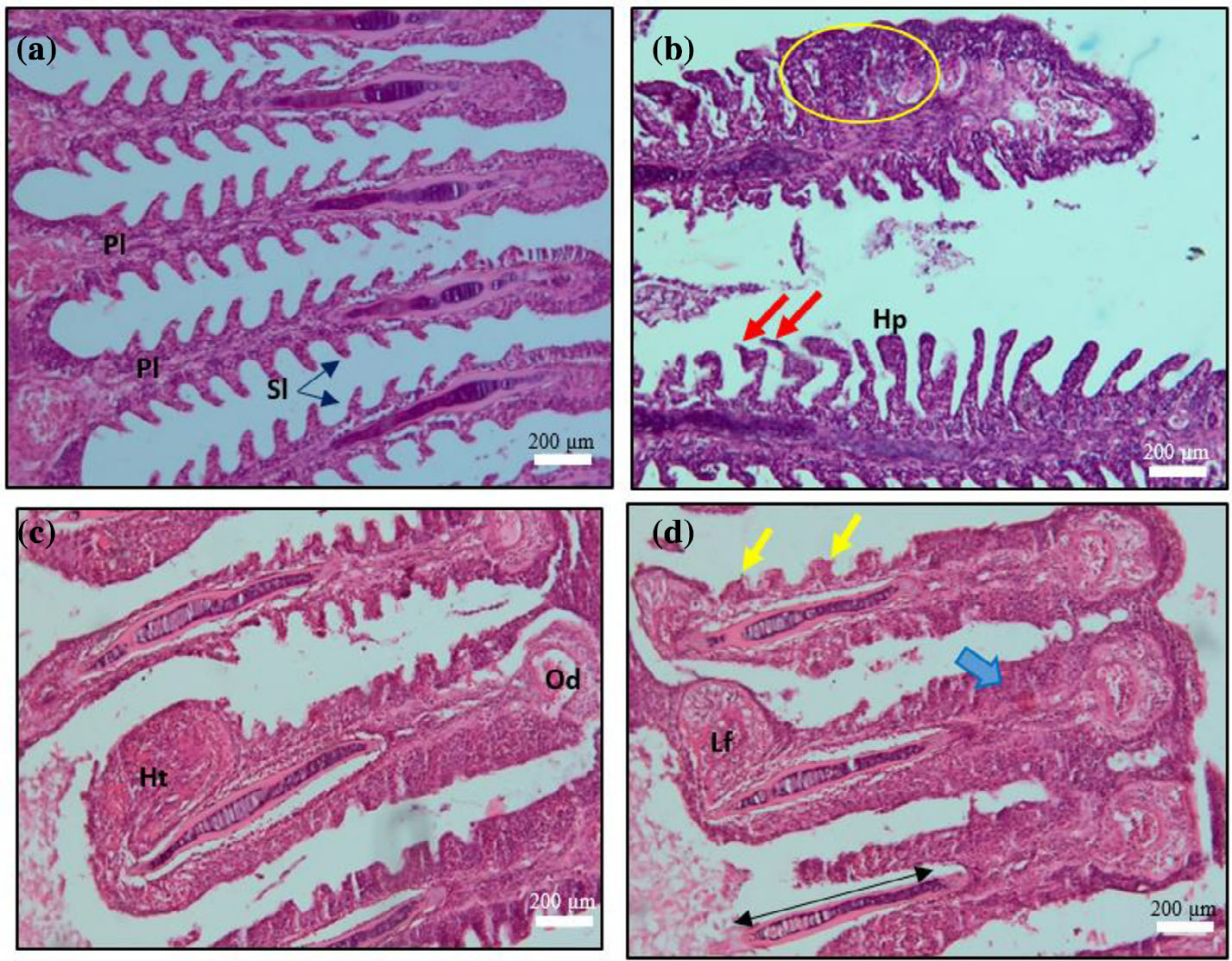
Histopathology of *Saprolegnia* spp. affected gill (a) primary lamellae (Pl), secondary lamellae (Sl) (b) fusion of secondary lamellae (circle), curved and ruptured secondary lamellae (red arrow), and hyperplasia (Hp) (c) hypertrophied primary gill lamella associated with fungal hyphae (Ht), oedema (thick arrow) (d) lamellar fusion (Lf), cartilaginous core (↔) containing cyst, clubbing (arrowhead), telangiectasis (circle), scale bar: 200 µm (H&E).

Gill tissues in the control group showed the most severe abnormalities in the organization of primary and secondary lamellae, with epithelial lifting, clubbing, oedema, hypertrophy, hyperplasia and other structural changes (Figure 4). In contrast, these primary histological abnormalities were resolved after 10 days of treatment in T4.

#### 3.7.3 Histopathology of Kidney

**FIGURE 5:**
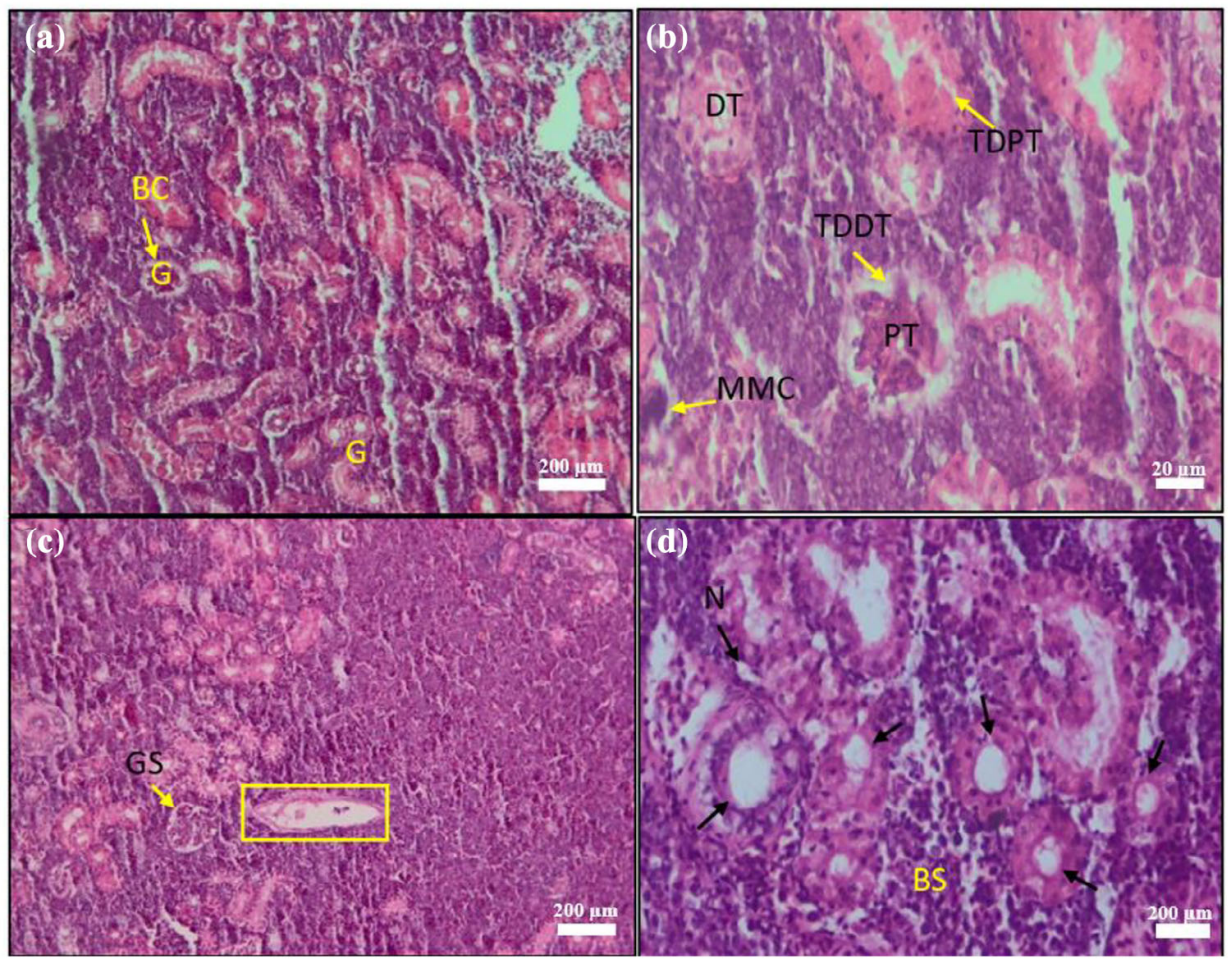
Histopathology of *Saprolegnia* spp.–affected kidney (a) glomerulus (G), Bowman's capsule (BC), (b) distal tubule (DT), proximal tubule (PT), melanomacrophage centres (MMCs), tubular degeneration of distal tubule (TDDT), tubular degeneration of proximal tubule (TDPT); (c) glomerular shrinkage (GS); (d) necrosis (N), vacuolation (→), Bowman's space (BS), scale bar: 20–200 µm (H&E).

Kidney sections in the first week (Figure 5) showed damage such as haematopoietic tissue loss, filtration space destruction, tubular degeneration, necrosis and leukocyte infiltration. By the trial's end, T3 and T4 groups exhibited reduced vascular anomalies, necrosis, and proximal tubule degeneration (Table 6).

#### 3.7.4 Histopathology of Liver

**FIGURE 6:**
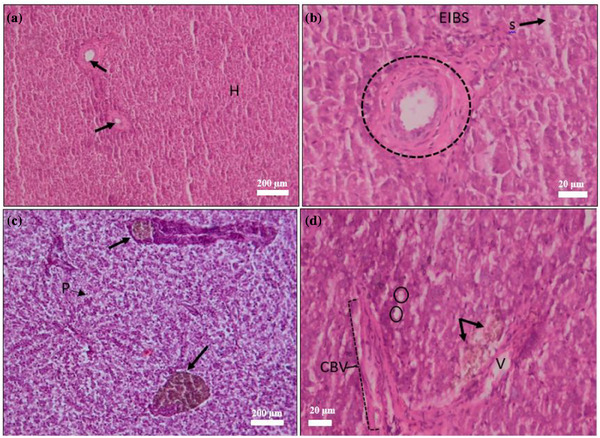
Histopathology of *Saprolegnia* spp. affected liver (a) hyperplasia (H), central vein (→); (b) higher magnification view of central vein (dotted circle), sinusoid cell (S), clumping of erythrocyte infiltration into blood sinusoid (EIBS); (c) pyknotic cell (P), melanomacrophage centre (thick arrow), sinusoid dilation (circle); (d) lipid droplets (circle), vacuolation (V), haemorrhage (→), congestion of blood vessel (CBV), scale bar: 20–200 µm (H&E).

Liver histopathology (Figure 6) revealed melanomacrophage centres (MMCs), hepatocyte necrosis, pyknotic nuclei and blood vessel congestion across treatments. At 10 days post‐treatment, the control group showed significant fatty hepatocyte deterioration, with large, distinct vacuoles replacing much of the cytoplasm.

#### 3.7.5 Histopathology of Spleen

Histological analysis of the spleen revealed intracellular oedema, ruptured spleen cells, expanded sinusoidal spaces and necrotic eosinophils across treatment groups. All infected groups exhibited white pulp loss and MMC activation. Notably, the T4 group showed complete elimination of these anomalies after 10 days of treatment.

### 3.8 Histopathological Scoring

After a 10‐day therapeutic trial, the results demonstrated varying degrees of histopathological changes across treatment groups in *C. punctata*. Fish in the control group (T1) exhibited the most severe tissue damage. The T2 group fishes (ivermectin + NaCl) showed moderate improvements, whereas T3 (CuSO_4_ + Viodin) resulted in further reductions in gill and skin abnormalities. Notably, the T4 group (increased temperature + NaCl) displayed the least severe histopathological alterations across all organs, with minimal necrosis and cellular degeneration.

### 3.9 Kaplan–Meier Survival Analysis

**FIGURE 7:**
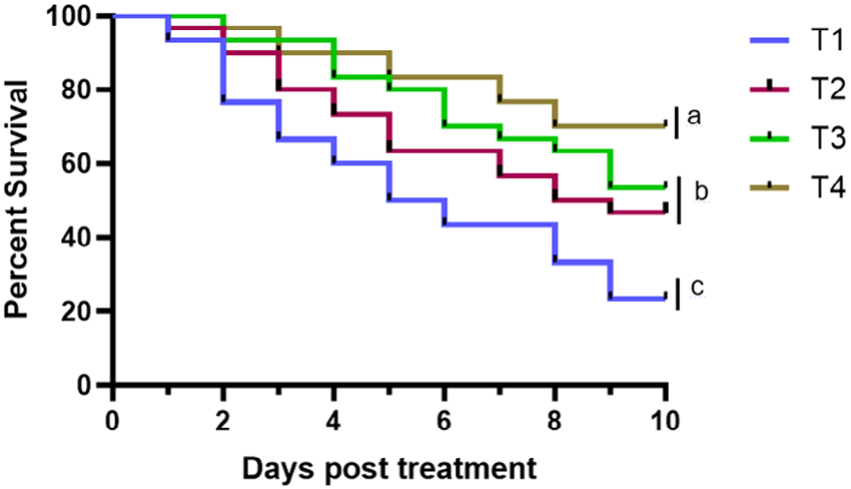
The Kaplan–Meier survival analysis for *Channa punctata* over a 10‐day treatment trial, showing significant differences in survival (*p* < 0.01) across treatment groups (*n* = 30 per group).

In the Kaplan–Meier survival analysis (Figure 7), T4 demonstrated the highest survival rate (*p* < 0.05) among the *C. punctata* population, reaching 70% over the 10‐day treatment period. In contrast, T1 showed the lowest survival rate, with a cumulative survival of only 23.33%. The survival rates in T3 (53.33%) and T2 (46.67%) were both significantly higher than in T1 (*p* < 0.05). These results indicate that T4 was the most effective treatment for improving survival rates, followed by T3 and T2, whereas T1 exhibited the highest mortality.

## 4 Discussion

**FIGURE 8:**
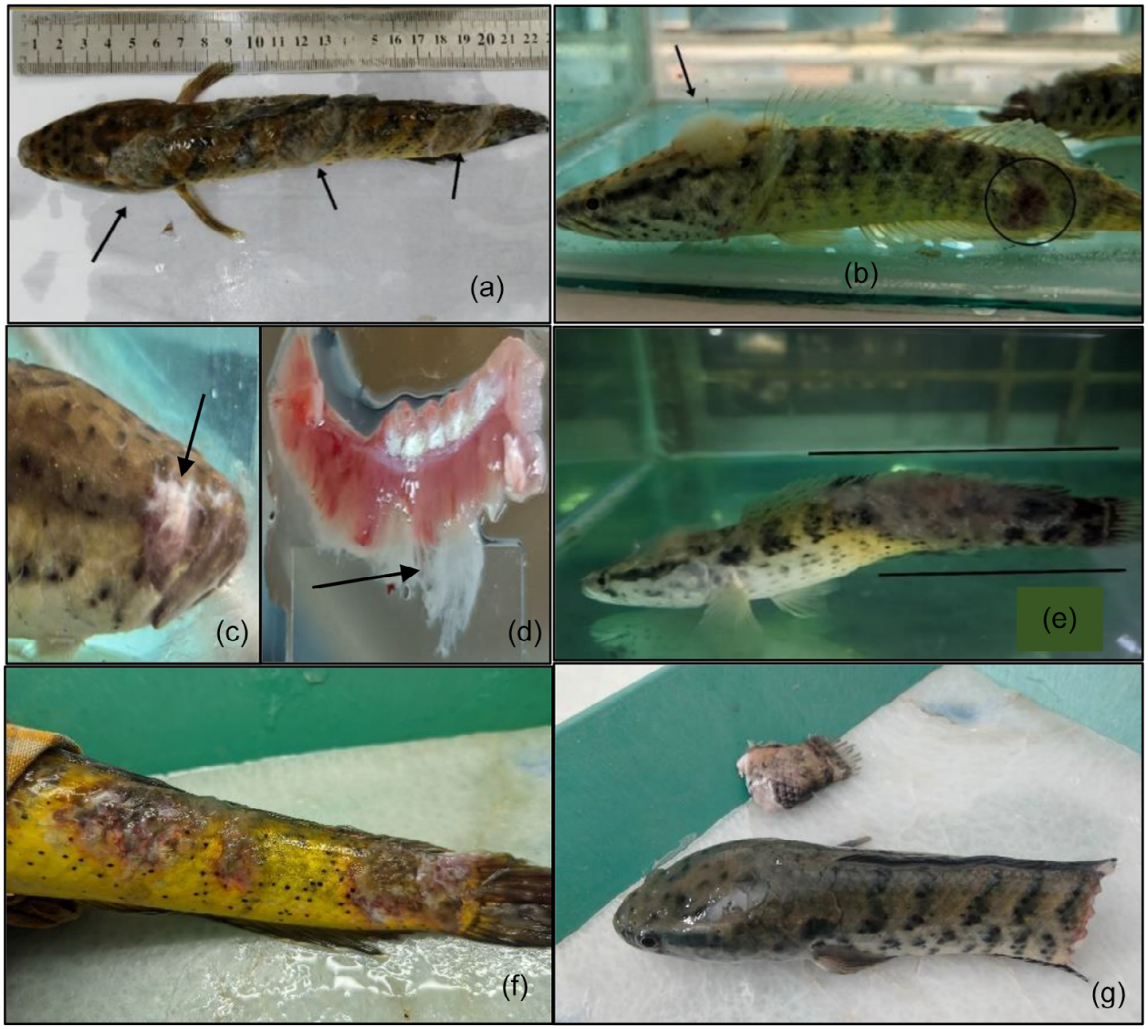
(a) Fluffy cotton–like white structure at the dorsal, caudal and pectoral regions (arrow). (b) Injuries at different parts of the body and fins. In some cases, the lesions encompassed 90% of their bodies. (c) Eye loss due to fungal attack. (d) Numerous fungal hyphae seen in infected fish gills. (e) Scoliosis in the body with a massive outbreak of fungal hyphae spread from dorsal region to caudal region. (f) Haemorrhage and several lesions or ulcers due to massive attack. (g) A live fish with a broken caudal fin, suspended in the water column.

**FIGURE 9:**
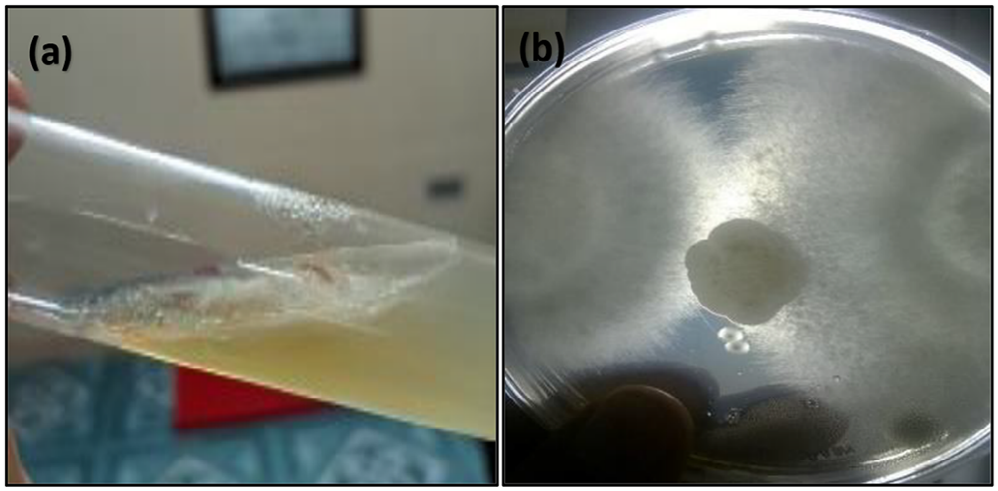
(a) *Saprolegnia* spp. mycelium on CDA slant; (b) plate culture method showing fungus isolated from infected spotted snakehead (*Channa punctata*).

The primary objective of this study was to evaluate the effectiveness of various treatment methods in preventing saprolegniasis. Additionally, our ongoing research sought to provide a comprehensive understanding of *Saprolegnia* spp. infections in spotted snakehead fish, focusing on a thorough examination of clinical manifestations, underlying causes, disease progression and detailed haematological and histological investigations. *Saprolegnia* spp. is a notorious fungal pathogen that poses significant threats to the aquaculture industry, leading to substantial economic losses. Saprolegniasis has caused high mortality rates across numerous fish species globally, including *Pseudochondrostoma duriense* (Aller‐Gancedo et al. 2016), *Salmo trutta* and *Thymallus thymallus* (Rocchi et al. 2017), *L. rohita* (Chauhan et al. 2014), *H. fossilis*, *M. cavasius* (Mastan 2015), *C. batrachus* (Mastan and Ahmad 2018), *C. carpio* (Magray et al. 2021) and murrels, *C. marulius* and *C. striata* (Olufemi 1985). The moribund fish, at the time of sampling, displayed severe necrotic infections; cotton wool–like structures on their body surfaces; fungal keratitis or fungal endophthalmitis; extensive damage to their dorsal and caudal fins; haemorrhages around the gills; and skin ulceration (Figure 8) were the primary clinical signs, which is consistent with previous narratives of saprolegniasis in a quite large number of fish species (Aller‐Gancedo et al. 2016; Tandel et al. 2021; Kumar et al. 2022; Elgendy et al. 2023). The gills were heavily infected with fungal hyphae as well. Fungal hyphae may extend to cover approximately 40%–50% of both the body surface and gills (Seymour 1970). Atlantic menhaden (*Brevoortia tyrannus*) and killifish (*Fundulus heteroclitus*) infected by *Aphanomyces invadans* were subjected to similar lesions and severe pathologies (Johnson et al. 2004). Samples of skin tissue lesions were cultured on CDA, displaying white cotton‐like mycelium, hair‐like features on spores, and aseptate hyphae typical of *Saprolegnia* spp. (Figure 9). After prolonged incubation, the hyphae thickened and spiralled, fully covering the plate. Similar observations were made with cultures on glucose peptone yeast agar, potato dextrose agar and Sabouraud dextrose agar (Lone and Manohar 2018; Kumar et al. 2020; Kumar et al. 2022; Elgendy et al. 2023).

Wet mount examination revealed branched aseptate hyphae, apical zoosporangia and oogonia (Figure 2), closely matched with previous descriptions (Sandoval‐Sierra et al. 2014; Zahran et al. 2017). A previous study also reported the presence of non‐septate hyphae with characteristic zoosporangia in wet mount preparations from skin lesions, confirming the identification of *Saprolegnia* spp. (Elgendy et al. 2023). The mycelia were larger and thicker than those of Ascomycetes and Basidiomycetes (Dieguez‐Uribeondo et al. 2004). In this study, temperatures ranged from 25°C ± 0.33°C to 30°C, consistent with the species tolerance up to 40°C (Courtenay and Williams 2004). Dissolved oxygen ranged from 5.67 ± 0.42 to 7.36 ± 0.26 mg/L, and pH from 7.48 ± 0.18 to 8.22 ± 0.11, within the tolerance range of 4.25–9.4 (Varma 1979; Boyd 1982). Ammonia levels varied from 3.14 ± 0.55 to 5.05 ± 0.18 due to high stocking density, but *C. punctata* showed no adverse effects (Marimuthu and Haniffa 2007). Nitrite levels were stable from 0.25 ± 0.04 to 0.35 ± 0.12, within acceptable limits (Boyd 1982).

Following the completion of the trial (on Day 10), blood samples were collected from the treated fishes to study the effect of different treatments on *Saprolegnia* spp. The findings revealed significant differences in haematological indices between treatments, indicating severe haematological abnormalities in the infected fish. Mycotic infection resulted in alterations in fish blood composition (Chauhan et al. 2014). Blood serves as a comprehensive reflection of the body's pathophysiology (Sharman and Singh 2004). Consequently, haematological parameters play a vital role in assessing the functional and physiological status of fish (Chagas and Val 2003). Notably, the affected fish had typical indications of anaemia, including a reduction in Hb content and red blood cell count (Table 5). The subsequent immunosuppression and haemodilution make it easier for diseases to spread quickly, which increases fish mortality. Furthermore, the presence of *Saprolegnia* spp. has previously been connected to the decrease in red blood cell count and Hb content in channel catfish (Durborow et al. 2003). Similar findings underscore the significant impact of *Saprolegnia* spp. on the haematological health of various fish species (Shah and Altindag 2004; Shah 2010). Numerous variables, including erythrocyte vulnerability, haemorrhage development, reduced WBC production and leukocyte destruction (Stueland et al. 2005), have been linked to haematological changes in a variety of fish species. The decrease in WBCs has been linked to increased haemodilution and corticosteroid production (Tort et al. 1987). In the current study, infected fish also showed enhanced WBC counts and a higher proportion of neutrophils and eosinophils as a result of the infection. In contrast, the proportion of lymphocytes and monocytes decreased, aligning with results from brown trout infected with *Saprolegnia* spp. (Alvarez et al. 1988).

Biochemical analysis of *Saprolegnia* spp.–infected *C. punctata* under different treatments revealed significant variations across multiple parameters, particularly in liver enzymes, protein metabolism and lipid profiles. Our findings align with those of Piasecki and Ostaszewska (2014), who investigated haematological and biochemical parameters in pikeperch infected with *Saprolegnia parasitica*, highlighting the specific effects of fungal pathogens on fish health. Similarly, El‐Matbouli and Soliman (2015) explored biochemical and haematological changes in fish infected with various parasites, offering broader insights relevant to understanding physiological responses in fungal infections like saprolegniasis.

**FIGURE 10:**
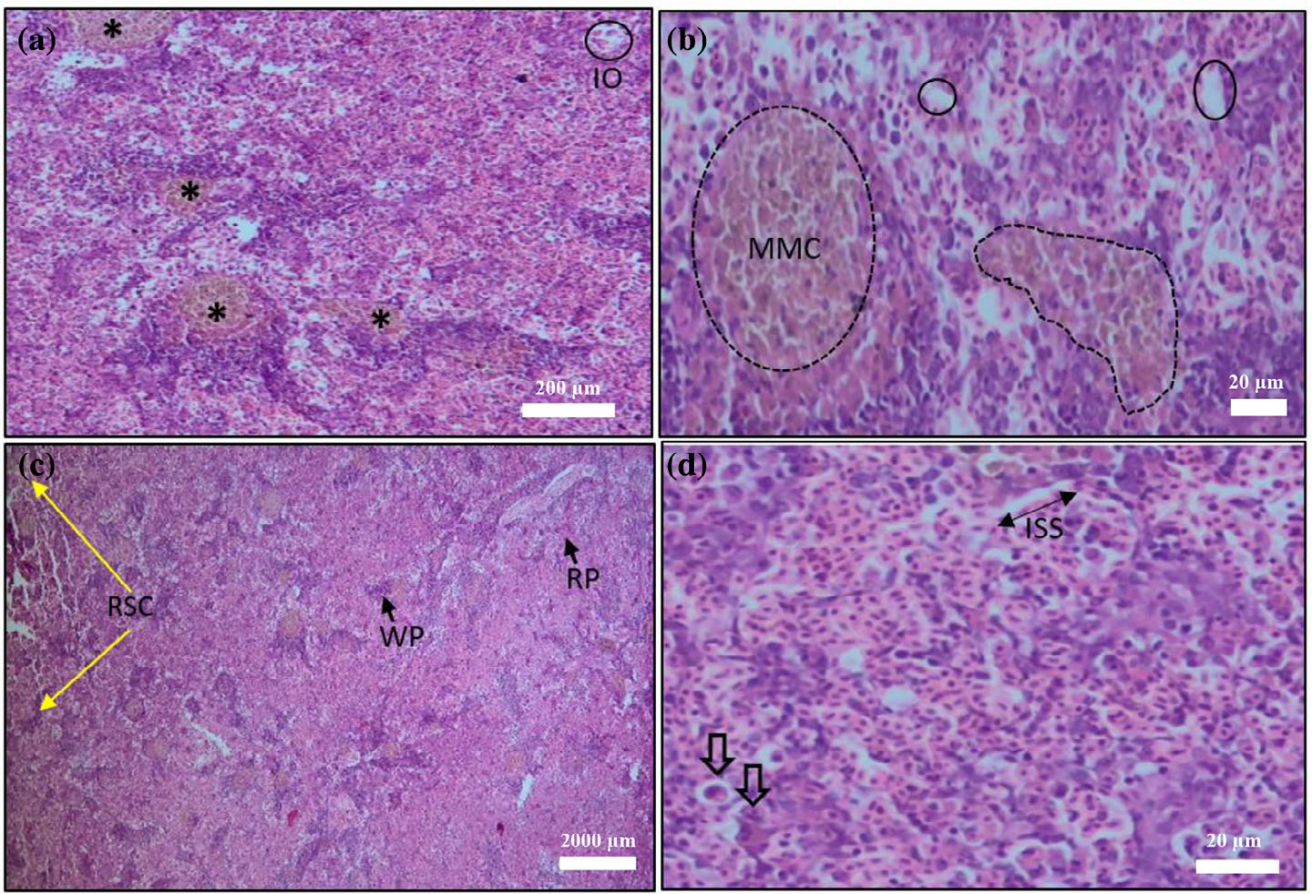
Histopathology of *Saprolegnia* spp. affected spleen (a) melanomacrophage centre (MMC) (*), intracellular oedema (IO); (b) higher magnification view of MMC (dotted circle), vacuole (small circle); (c) raptured spleen cell (RSC), white pulp (WP), red pulp (RP); (d) increase of sinusoidal space (ISS), necrotic eosinophil (thick arrow), scale bar: 20–2000 µm (H&E).

At the end of the treatment trial, histopathological analysis revealed significant alterations across multiple organs, including the skin, gills, kidney, liver, spleen and muscle of *C. punctata* infected with *Saprolegnia* spp. (Figures 3, 4, 5, 6, 10). Histopathological indicators, including sloughed‐off epidermis, necrosis, ulcers and scale damage, highlighted the invasive potential of the pathogen (Amer et al. 2020; Zaman et al. 2023). Similar findings have been noted in *Saprolegnia* spp.–infected zebrafish (Hussein et al. 2013), common carp (Ashour et al. 2017) and rainbow trout (Shin et al. 2017). Remarkably, fish in the T4 treatment group displayed improved skin and gill conditions, with increased mononuclear cells and shedding of infected epidermal layers, suggesting effective fungal clearance. Gill tissue abnormalities included epithelial hyperplasia, sub‐epithelial oedema and lamellae curling, consistent with prior reports in *C. carpio* (Ashour et al. 2017) and tilapia (Hamad and Mustafa 2018). Liver histopathology indicated erythrocyte infiltration, MMCs and sinusoid dilatation, aligning with findings in *Clarias gariepinus* (Olojo et al. 2005) and *Oncorhynchus mykiss* (Atamanalp et al. 2008; Refai et al. 2008). Additionally, splenic tissue showed ruptured cells, necrotic eosinophils and intracellular oedema, comparable to observations in brown trout infected with *S. parasitica* (Alvarez et al. 1988). Kidney histopathology also revealed tubular necrosis and MMCs, mirroring previous studies in rainbow trout, common carp and European eels (Sarowar et al. 2014; Pottinger and Day 1999; Yardimci and Turgay 2020). Overall, T4 treatment displayed reduced severity of histopathological changes in the visceral organs, underscoring its therapeutic potential.

Various chemicals have been employed for the prevention and treatment of *Saprolegnia* spp., such as malachite green, formalin (Willoughby and Roberts 1992), 37% concentrated formaldehyde solution (Fitzpatrick et al. 1995; Schreier et al. 1996), hydrogen peroxide (Speare and Arsenault 1997; Howe et al. 1999; Derksen et al. 1999) and iodine‐free salt (NaCl), bronopol (Pottinger and Day 1999). However, those chemical treatments often applied on a temporal basis are also not particularly effective. Because *Saprolegnia* spp. infections are widespread and have a high rate of dissemination in aquatic habitats, eliminating the disease is challenging. The present study is the first to demonstrate the therapeutic effects of various treatments against saprolegniasis in *C. punctata*. Increasing NaCl concentrations have been shown to reduce *Saprolegnia* spp. growth rates on agar medium (Sformo et al. 2021), a trend that was also observed in this study. Treatments combining saline (salt) with ivermectin (T2) and CuSO_4_ with iodine (T3) did not significantly reduce the fungal burden of white cotton‐like hyphae in *C. punctata*, as seen in the Kaplan–Meier survival analysis (Figure 7), where cumulative survival rates remained below 55% for both treatments. However, the survival rate in T2 and T3 was significantly higher than the control group, which experienced the highest mortality, with a survival rate of only 23%. In contrast, T4 showed a significantly higher survival rate of 70% (*p* < 0.05) compared to all other treatments. Eissa et al. (2013) reported that rinsing angelfish spawners and eggs with povidone‐iodine (60–70 mg/L) significantly reduced fungal infections, decreasing egg mortality from 70% to 30% and increasing hatching rates from 10% to 60%, confirming its effectiveness as a hatchery disinfectant. Salt has been recognized as an effective and widely used preventative measure for *Saprolegnia* spp. infections in freshwater aquaculture (Francis‐Floyd et al. 1993; van West 2006). Additionally, *Saprolegnia* spp. does not grow in vitro at temperatures above 30°C (Koeypudsa et al. 2005), supporting our findings. Furthermore, manipulating water temperature in glass aquariums has been highly effective in controlling protozoan diseases such as *Ichthyophthirius multifiliis* in both food and ornamental fish species (Mamun et al. 2020; Mamun et al. 2021). Therefore, elevating temperature with salt emerges as a viable option for treating *Saprolegnia* spp. infections in snakehead, *C. punctata*. Although the combined application of elevated temperature (30°C) and 2% NaCl demonstrated promising therapeutic effects under controlled conditions, we recognize that implementing such protocols at a commercial scale may be challenging. Field environments often lack the infrastructure to precisely regulate temperature or salinity, especially during large‐scale outbreaks. Therefore, further field‐based research is crucial to validate the comprehensive understanding of the treatment strategies for *Saprolegnia* spp. infections in *C. punctata* and their practical implications in aquaculture.

## 5 Conclusion

This study demonstrates that managing *Saprolegnia* spp. infections in *C. punctata* can be effectively achieved through targeted temperature and salinity control, both of which significantly enhance fish health and survival. Haematological, biochemical and histopathological analyses confirm the physiological benefits of these treatments, showing reduced infection impacts and improved resilience in treated fish. Further research and practical application of these methods could support sustainable fish farming practices, minimizing economic losses and enhancing the well‐being of fish populations.

## Author Contributions

Md. Abdullah Al Mamun led the conceptualization, validation and project administration and contributed to funding acquisition and writing the original draft. Md. Shaif Rahman handled the methodology, software, formal analysis and contributed to writing the original draft. Md. Siddikur Rahman Sujon contributed to the methodology, software, data curation, investigation and visualization. Susmita Roy assisted with methodology and data curation. Shamima Nasren supervised the project and contributed to funding acquisition, writing reviews and editing. Tofael Ahmed Sumon and Sarker Md. Ibrahim Khalil provided resources and contributed to writing reviews and editing. M. M. Mahbub Alam contributed to visualization and writing review and editing.

## Ethics Statement

The Animal Ethics Committee of Sylhet Agricultural University provided guidelines for the handling of fish in this study and use of antibiotics (Memo: SAU/AEC/FOF/FHM‐513), which were strictly followed.

## Conflicts of Interest

The authors declare no conflicts of interest.

## Peer Review

The peer review history for this article is available at https://www.webofscience.com/api/gateway/wos/peer‐review/10.1002/vms3.70473.

## Data Availability

Data are available from the corresponding author on request.
